# Factors That Influence Non-Motor Impairment Across the ALS-FTD Spectrum: Impact of Phenotype, Sex, Age, Onset and Disease Stage

**DOI:** 10.3389/fneur.2021.743688

**Published:** 2021-11-25

**Authors:** Emma M. Devenney, Kate McErlean, Nga Yan Tse, Jashelle Caga, Thanuja Dharmadasa, William Huynh, Colin J. Mahoney, Margaret Zoing, Srestha Mazumder, Carol Dobson-Stone, John B. Kwok, Glenda M. Halliday, John R. Hodges, Olivier Piguet, Rebekah M. Ahmed, Matthew C. Kiernan

**Affiliations:** ^1^Brain and Mind Centre, The University of Sydney, Sydney, NSW, Australia; ^2^Faculty of Medicine and Health, The University of Sydney, Sydney, NSW, Australia; ^3^Royal College of Surgeons in Ireland, Dublin, Ireland; ^4^Nuffield Department of Clinical Neurosciences, University of Oxford, Oxford, United Kingdom; ^5^Prince of Wales Clinical School, University of New South Wales, Kensington, NSW, Australia; ^6^School of Psychology, The University of Sydney, Sydney, NSW, Australia; ^7^Institute of Clinical Neurosciences, Royal Prince Alfred Hospital, Camperdown, NSW, Australia

**Keywords:** ALS (amyotrophic lateral sclerosis), behavioral impairment, non-motor deficits, neuropsychiatric symptoms, frontotemporal dementia

## Abstract

**Objective:** This study aimed to establish (1) the pattern and severity of neuropsychiatric symptoms and other non-motor symptoms of sleep and mood, across ALS phenotypes in comparison to bvFTD and (2) the contribution of non-modifiable factors including age, sex and disease state to the severity of symptoms experienced by ALS patients.

**Methods:** Consecutive participants were recruited to the study and underwent a detailed clinical, cognitive, behavioral and neuroimaging assessment. Neuropsychiatric and other non-motor symptoms were determined using the Cambridge Behavioral Inventory, the CBI-R. The scores were converted to define impairment in terms of mild, moderate and severe symptoms for each subscale. Rate, severity and contribution of King's staging and modifiable factors were also determined and a regression model identified predictors of symptom severity.

**Results:** In total, 250 participants (115 ALS, 98 bvFTD, and 37 ALS-FTD patients) were recruited. A similar pattern of neuropsychiatric symptom severity was identified (apathy, disinhibition and stereotypic behavior) for all behavioral phenotypes of ALS compared to bvFTD (all *p* > 0.05). Neuropsychiatric symptoms were also present in cases defined as ALSpure and the cognitive phenotype of ALS (ALSci) although they occurred less frequently and were at the milder end of the spectrum. Disordered sleep and disrupted mood were common across all phenotypes (all *p* < 0.05). The severity of sleep dysfunction was influenced by both sex and age (all *p* < 0.05). Neuropsychiatric symptoms, sleep and mood disorders were common early in the disease process and deteriorated in line with progression on the Amyotrophic Lateral Sclerosis Functional Rating Scale-Revised (ALSFRS-R; all *p* < 0.05). Diagnostic phenotype, disease duration and global cognition scores were the strongest predictors of non-motor and neuropsychiatric impairments.

**Conclusion:** The current findings reveal strikingly similar patterns of changes across the subgroups of ALS and bvFTD, supporting the concept of the ALS-FTD spectrum. The findings further highlight the impact of non-motor and neuropsychiatric symptoms in patients with ALS, that are often as severe as that seen in ALS-FTD and bvFTD. This study advances understanding across the ALS-FTD spectrum that may accelerate the early identification of patient needs, to ensure prompt recognition of symptoms and thereby to improve clinical awareness, patient care and management.

## Introduction

Amyotrophic lateral sclerosis (ALS) is a relentlessly progressive neurodegenerative disease that leads to death within 3–5 years of disease onset (1, 2). The conceptualisation of this disorder has changed dramatically over recent decades from one that was traditionally considered to be a purely motor disorder, to what is now considered a multisystem neurodegenerative condition. Cognitive and behavioral impairments, similar to those seen in Frontotemporal Dementia (FTD), are common. Indeed, some develop frank FTD while the majority demonstrate less marked impairment (3–5). As such, ALS and FTD appear to exist on a spectrum with the overlap extending beyond cognition and behavior to encompass genetic, pathological, clinical and neuroimaging features (2).

Consensus criteria for the diagnosis of ALS with associated cognitive and behavioral impairment have recently been updated (6). The designation of behavioral impairment relies heavily on the presence of apathy, which is perhaps not surprising given that of all the behavioral impairments, apathy or loss of motivation toward goal-directed behavior is most commonly reported, occurring in 30–60% of ALS cases depending on the criteria applied (3, 7). Behavioral impairment can also be diagnosed if two other abnormal behaviors are present reflecting the overlapping phenotype with FTD, these include stereotypic behavior, disinhibition and impulsivity, changes in dietary preferences and loss of empathy (8).

Neuropsychiatric symptoms in ALS have generally received less attention than cognitive dysfunction and to date there have been few studies that have compared across the spectrum from FTD to ALS with none at the level of granularity of cognitive and behavioral subtypes (3, 9). Across the neuropsychiatric symptoms, apathy has received the most scrutiny. The presence of apathy predicts a more aggressive disease progression, reduced quality of life and leads to issues with treatment compliance. This serves as an exemplar of the impact of neuropsychiatric impairment in ALS and the need to better understand and quantify the impact of these behaviors across ALS subtypes (10–12). Whether behavioral impairment follows the typically relentless and progressive disease trajectory of ALS, has been debated, but recent evidence from a large multi-centre study suggests that dysfunctional behaviors do progress over time (13). Non-Motor impairment outside that of behavioral and cognitive change, encompasses disordered sleep, changes in mood including depression and anxiety symptoms, pain and fatigue (14–17). These features are becoming increasingly recognized as core components of the disorder and may be related to underlying extra-motor cortical spread of pathology, associated with a worse prognosis (18, 19).

Analysing and predicting patterns of non-motor dysfunction represents an important aspect of disease management in terms of patient expectation, carer burden, disease progression and treatment options. Educating those affected by ALS and being able to manage expectations regarding neuropsychiatric symptoms is invaluable to caregivers and patients. While much knowledge has been gained recently regarding the overlap across FTD and ALS, the extent of similarities (or differences) across this spectrum in terms of patterns of non-motor impairment remains less well-explored. In addition it is not well understood how these symptoms are influenced by non-modifiable factors. In light of this, the present study aims to (1) identify the pattern of non-motor impairment across the ALS cohort compared to bvFTD; (2) determine the overlapping features across ALS subtypes to ultimately confirm the extent of the ALS-FTD spectrum; and finally (3) establish the extent that non-modifiable factors including cognitive function and disease duration impact on the severity of behaviors.

## Materials and Methods

### Participants

In total, 250 participants (115 ALS, 98 bvFTD, and 37 ALS-FTD patients) were recruited from the FOREFRONT ALS and FTD, and the FRONTIER FTD, multidisciplinary research clinics specializing in younger-onset dementias and motor neurodegenerative syndromes at the Brain and Mind Centre, University of Sydney in Australia. Standard diagnostic assessment consisted of: a medical and neurological examination, neuropsychological assessment, and clinical interviews with patients and carers. When ALS was suspected, neurophysiological examination was conducted including nerve conduction studies and transcranial magnetic stimulation, the latter used to identify features of upper motor neurone dysfunction (20, 21). Diagnosis was determined by multidisciplinary consensus in accordance with the current clinical diagnostic criteria for ALS (22–24), bvFTD (8) and ALS-FTD (6). Based on the Strong et al. criteria, all ALS patients were further sub-classified into ALS with no cognitive or behavioral impairment (i.e., ALSpure; *n* = 64), ALS with cognitive impairment (i.e., ALSci; determined by the presence of impaired letter fluency; *n* = 23), ALS with behavioral impairment (i.e., ALSbi; based on the informant-report Motor Neuron Disease behavior Scale [MiND-B]; *n* = 16), or ALS with combined cognitive and behavioral impairment (i.e., ALScbi; *n* = 12) (6, 25). ALS clinical stage was estimated using the King's Staging Criteria ranging from 1 to 4 increasing in line with functional impairment with a stage of 4b denoting respiratory impairment while 1–3 represents a cumulative score of body regions (bulbar, lower limb, upper limb) involved (26, 27). Exclusion criteria for all participants included the presence of other significant neurological or neurodegenerative syndrome. No patients required non-invasive ventilation at the time of the study.

### Blood Sampling

All patients and controls underwent blood sampling to screen for a *C9orf72* repeat expansion. Genomic DNA was extracted from peripheral blood lymphocytes. Proband DNA samples were then screened for the hexanucleotide repeat expansion in the *C9orf72* gene using a repeat primed polymerase chain reaction based on the protocol of Renton et al. (28). Samples were scored as positive if they harbored an allele with more than 30 repeats.

### Cognitive and Behavioral Measures

The third edition of the Addenbrooke's Cognitive Examination (ACE-III) was administered to measure overall cognitive functioning with a total score of <88 indicative of the presence of cognitive impairment (29). Performance on the letter fluency subdomain (i.e., the number of words beginning with the letter P generated within one min—adjusted for bulbar dysfunction based on validated fluency index for ALS) was used to determine letter fluency impairment defined as a score of 1.5 standard deviations or more below the previously reported healthy control mean.

To determine the presence of behavioral impairments in ALS patients, carers of patients completed the MiND-B that is designed to measure three domains of behavioral impairments: disinhibition (defined as a subdomain score of <13), apathy (a subdomain score of <9) and stereotypic behavior (a subdomain score of <5).

To comprehensively capture the pattern of non-motor symptoms, carers completed the revised Cambridge behavioral Inventory (CBI-R) (30). For the purpose of this study those domains that reflected the behavioral impairment required for a diagnosis of bvFTD were examined, in addition to those that represented non-motor impairments consisting of abnormal behavior (i.e., disinhibition), abnormal eating habits, stereotypic behaviors (i.e., perseverative and ritualistic behaviors), and reduced motivation (i.e., apathy and inertia), mood and sleep changes.

### Statistical Analyses

Data were analyzed using SPSS Statistics, version 26.0 (IBM, Armonk, NY). The statistical significance level was set at *p* < 0.05 for all analyses unless otherwise specified. One-way analysis of variance (ANOVA) was used to examine differences in demographic (i.e., age and education years), clinical (i.e., disease duration) and cognitive (i.e., ACE total score) variables between all groups (i.e., ALSpure, ALSci, ALSbi, ALScbi, bvFTD and ALS-FTD), followed by Sidak *post-hoc* tests. In the case of violation of heterogeneity of variance based on Levene's test results, Welch's *F* was used and followed by Games–Howell *post-hoc* tests. Categorical variables (i.e., sex, *C9orf72* expansion status, site of onset and King's stage) was examined using chi-squared tests.

To determine the rate of symptoms associated with each diagnostic group (i.e., ALSpure, ALSci, ALSbi, ALScbi, bvFTD and ALS-FTD), all non-motor subdomain scores were converted into categories of different severities of impairment: 0% = no symptoms, 1–25% = mild; 26–50% = moderate; 51–75% = severe; and >75% = very severe in accordance with previous literature (7). Following which, the percentage of patients with symptoms of at least mild and moderate severity were calculated for the six subdomains reflecting behavioral changes commonly associated with ALS-FTD spectrum and non-motor symptoms (i.e., abnormal behavior, mood changes, abnormal eating habits, sleep changes, stereotypic behaviors, and reduced motivation), respectively.

Within the ALS subgroups, to determine the prevalence of symptoms associated with each clinical stage (i.e., King's stage 1, 2, 3 and 4) and site of onset (i.e., bulbar and limb onset), all non-motor subdomain scores were converted into categories of either absence (i.e., a score of 0) or presence of symptoms (i.e., any score > 0). Next, the distribution of ALS patients by presence of symptoms was compared between clinical stages and site of onset, respectively, using chi-squared tests.

ANOVA was performed to examine differences in the six subdomain scores between diagnosis groups. To further explore whether differences exist in the severity of behavioral symptoms in those demonstrating behavioral symptoms between disease groups, the subdomain scores of those rated as presenting with at least mild severity of symptoms were compared between clinical groups within the six subdomains separately.

Lastly, to account for the contribution of cognitive and clinical variables to the severity of overall behavioral disturbance, a multiple linear regression was conducted to evaluate the degree to which diagnostic group, *C9orf72* expansion status, ACE-III total scores, education level and disease duration predicted total score on the CBI-R.

## Results

### Demographics

As expected, disease duration was longer in patients with bvFTD, compared with the ALS phenotypes (all *p* < 0.05). Similarly, patients with bvFTD and ALS-FTD showed more global cognitive impairment, as demonstrated on the ACE-III, than those without frank dementia (all *p* < 0.01). No significant group differences were found in age or sex distribution across all groups, (*p* > 0.05; Table 1). Educational level differed significantly with ALSpure and ALSbi groups (both *p* = 0.04) demonstrating a significant higher level of education compared to ALScbi groups. No significant difference was found for rate of the *C9orf72* expansion across groups (*p* > 0.05).

**Table 1:** Demographic characteristics across the spectrum of ALS-FTD.

	**ALSpure (*n* = 64)**	**ALSci(*n* = 23)**	**ALSbi (*n* = 16)**	**ALScbi(*n* = 12)**	**bvFTD (*n* = 98)**	**ALS-FTD(*n* = 37)**	***F***	***p***	***Post-hoc***
Sex (M/F)	33/31	13/10	10/6	5/7	61/37	27/10	6.499^a^	0.261	-
Age (Y)	61.54 (9.91)	59.83 (9.71)	60.81 (9.53)	66.75 (11.55)	62.98 (8.66)	63.84 (7.96)	1.356	0.241	-
Education (Y)	13.52 (3.30)	12.35 (2.90)	14.13 (3.81)	10.42 (3.12)	12.22 (2.94)	12.28 (3.40)	3.333	0.006	ALSpure,ALSbi > ALScbi
Disease duration (M)	26.44 (30.98)	46.65 (80.01)	32.25 (22.12)	23.67 (18.58)	53.48 (36.17)	33.89 (22.72)	6.723^b^	<0.001	bvFTD > ALSpure, ALSbi, ALScbi, ALS-FTD
Onset Site (Bulbar/Limb)	25/39	10/13	5/11	7/5	-	-	2.278^a^	0.517	-
*C9orf72* status (Y/N)	9/44	3/18	1/12	1/9	13/77	7/24	2.199^a^	0.821	-
King's staging (1/2/3/4)	32/14/14/4	9/6/8/0	4/3/7/2	2/4/4/2	-	-	11.481^a^	0.244	-
ACE Total (/100)	92.19 (5.81)	86.52 (8.21)	91.73 (7.70)	75.17 (8.97)	66.07 (20.73)	60.70 (18.10)	48.624^b^	<0.001	All ALS groups > ALS-FTD & bvFTD
									ALSpure > ALScbi

*Means (Standard Deviation)*.

a *Chi-square value*.

b *Welch's F value*.

*ACE, Addenbrooke's Cognitive Examination; ALSbi, amyotrophic lateral sclerosis with behavioral impairment; ALSci, amyotrophic lateral sclerosis with cognitive impairment; ALScbi, amyotrophic lateral sclerosis with combined cognitive and behavioral impairment; ALSpure, amyotrophic lateral sclerosis with no cognitive or behavioral impairment; ALS-FTD, amyotrophic lateral sclerosis-frontotemporal dementia-motor neuron disease; bvFTD, behavioral-variant frontotemporal dementia*.

Within the ALS subgroups, no significant differences were observed in site of onset or clinical stage (determined in accordance with King's staging criteria; *p* > 0.05; Table 1).

### Frequency of Non-Motor Disturbances Across ALS-FTD Phenotypes

#### Impairment Across all Levels of Severity

**Figure 1:**
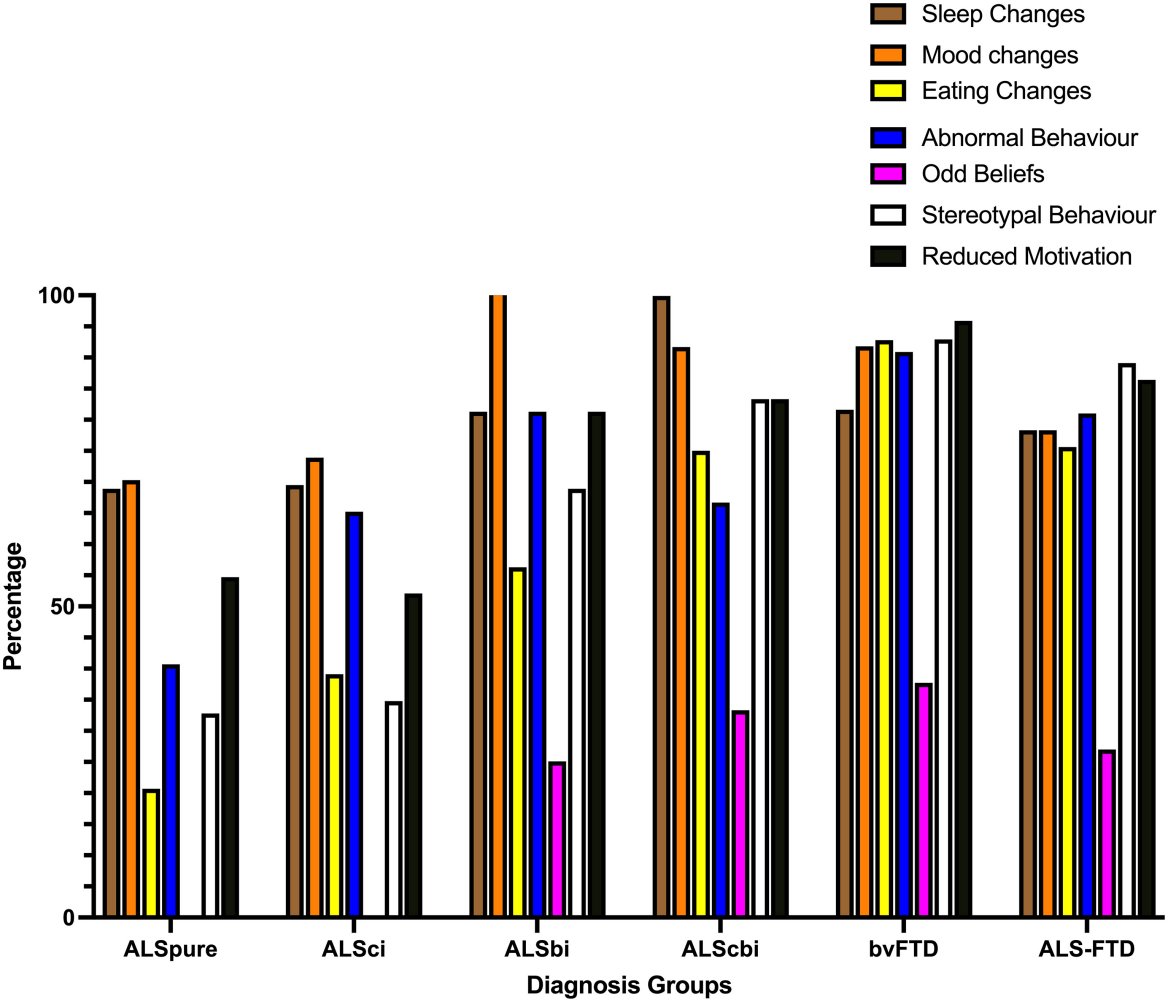
Bar chart displaying frequency of behavioral symptoms of at least mild severity on the revised Cambridge behavioral Inventory. ALSbi, amyotrophic lateral sclerosis with behavioral impairment; ALSci, amyotrophic lateral sclerosis with cognitive impairment; ALScbi, amyotrophic lateral sclerosis with combined cognitive and behavioral impairment; ALSpure, amyotrophic lateral sclerosis with no cognitive or behavioral impairment; bvFTD, behavioral-variant frontotemporal dementia; ALS-FTD, amyotrophic lateral sclerosis-frontotemporal dementia.

In terms of the frequency of impairments (at any severity level), ~70% of ALSpure patients demonstrated mood and sleep changes (see Figure 1), followed by reduced motivation (54.7%), abnormal (40.7%) and stereotypic (32.8%) behavior, and eating changes (20.7%).

In ALSci, mood (73.9%) and sleep (69.5%) changes similarly were the most common behavioral disturbances, however, more than half also demonstrated abnormal behavior (65.2%) and reduced motivation (52.1%), followed by eating changes (39.1%) and stereotypic behavior (34.8%).

All ALSbi patients were rated by their carer as demonstrating mood changes (100%), and ~80% of them experienced reduced motivation, abnormal behavior and sleep changes, followed by stereotypic behavior (68.9%) and eating changes (56.3%).

In ALScbi, sleep (99.9%) and mood (91.7%) changes occurred in nearly all the patients, while 83.3% experienced stereotypic behavior and reduced motivation, followed by eating changes (75%) and abnormal behavior (66.7%).

A high prevalence of reduced motivation, stereotypic behavior, eating and mood changes, sleep changes and abnormal behavior were reported in bvFTD (all >80%).

#### Moderate to Severe Impairment

**Figure 2:**
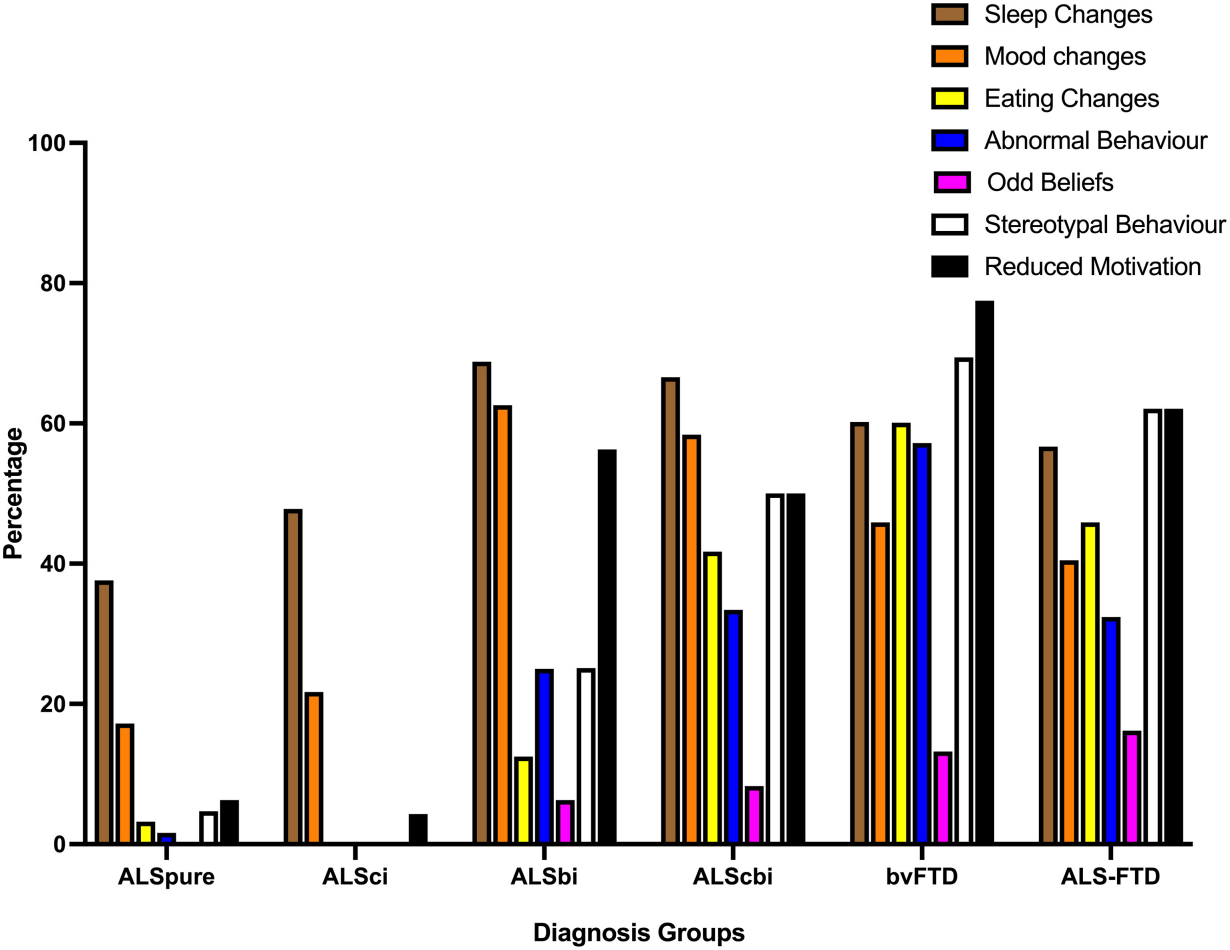
Bar chart displaying frequency of behavioral impairments of at least moderate severity on the revised Cambridge behavioral Inventory. ALSbi, amyotrophic lateral sclerosis with behavioral impairment; ALSci, amyotrophic lateral sclerosis with cognitive impairment; ALScbi, amyotrophic lateral sclerosis with combined cognitive and behavioral impairment; ALSpure, amyotrophic lateral sclerosis with no cognitive or behavioral impairment; bvFTD, behavioral-variant frontotemporal dementia; ALS-FTD, amyotrophic lateral sclerosis-frontotemporal dementia.

Considering behavioral impairments of at least moderate severity, sleep and mood changes were the most commonly reported behavioral symptoms across all ALS subgroups (see Figure 2). In both the ALSbi and ALScbi groups reduced motivation was also reported in ≥50%, whilst abnormal and stereotypic behavior was demonstrated in 25% (ALSbi) and >50% (ALScbi) Additionally, a further 41.7 and 33.4% of ALScbi patients demonstrated eating changes and abnormal behavior, respectively. Conversely, stereotypic and reduced motivation (>62%) were the most common behavioral disturbances of moderate-severe severity in bvFTD and ALS-FTD.

#### Comparison of ALS-FTD Phenotypes

By comparing frequency (of all severities) across groups there were no significant differences in the rate of sleep disorders (all *p* > 0.05), while mood differed only for bvFTD compared to ALSPure (*p* = 0.001).

In comparison, there was a clear spectrum of increasing frequency of neuropsychiatric symptoms from ALSpure to bvFTD for stereotypy, motivation, eating and abnormal behavior.

In more detail, ALSbi (*p* = 0.002), ALScbi (*p* = 0.001), bvFTD and ALS-FTD (both *p* < 0.001) experienced stereotypy more frequently than ALSpure, while ALSci experienced stereotypy less frequently than bvFTD and ALS-FTD (both *p* < 0.001).

Similarly, for abnormal behavior, ALSbi (*p* = 0.004), bvFTD and ALS-FTD (*p* < 0.001) endorsed abnormal behavior more frequently than ALSpure, while ALSci experienced abnormal behavior less frequently than bvFTD and ALS-FTD (both *p* = 0.001).

In the domain of motivation, changes were less frequently endorsed by carers of patients with ALSpure and ALSci endorsed changes in motivation less frequently than in bvFTD (both *p* < 0.001) and ALS-FTD (*p* = 0.001 and 0.006 respectively) but with comparable frequencies to ALSbi, ALSci and ALScbi (all *p* > 0.09).

Finally, eating changes were endorsed more frequently by carers of patients with ALScbi, ALS-FTD and bvFTD than ALSpure (all *p* < 0.001), while eating changes were more common in bvFTD than ALSbi (*p* = 0.001) and more common in bvFTD and ALS-FTD than ALSci (*p* < 0.001 and *p* = 0.007 respectively).

### Severity of Symptoms Across ALS-FTD Phenotypes

A greater severity of eating changes, abnormal behavior, stereotypic behavior and reduced motivation were demonstrated in bvFTD and ALS-FTD groups relative to ALSpure and ALSci groups (all *p* < 0.05; Table 2). bvFTD was further revealed to show a greater extent of impairment in abnormal and stereotypic behavior compared to ALSbi group (all *p* < *0.0*5). In contrast, comparable level of mood changes were observed with only the bvFTD group demonstrating greater mood symptoms compared to ALSpure group (*p* = 0.048). No significant differences were found in sleep changes between all groups.

**Table 2:** Severity of neuropsychiatric and non-motor symptoms between groups across the ALS-FTD spectrum.

	**ALSpure**	**ALSci**	**ALSbi**	**ALScbi**	**bvFTD**	**ALS-FTD**	***F***	***p***	***Post-hoc***
Mood changes	21.47 (14.23)	21.94 (15.83)	36.72 (15.12)	34.09 (20.23)	31.16 (20.29)	30.60 (16.98)	3.217	0.008	bvFTD > ALSpure
Sleep changes	38.64 (24.53)	30.11 (26.35)	46.67 (31.15)	50.00 (27.18)	41.84 (31.15)	36.49 (28.16)	1.174	0.323	-
Eating changes	19.23 (15.50)	11.81 (6.59)	23.61 (13.18)	28.47 (15.34)	43.57 (26.88)	38.02 (23.05)	16.781^a^	<0.001	bvFTD, ALS-FTD > ALSpure, ALSci
									bvFTD > ALSbi
Abnormal behaviors	10.58 (6.99)	11.39 (7.63)	23.40 (12.45)	28.13 (26.70)	38.24 (22.99)	29.28 (20.00)	22.828^a^	<0.001	bvFTD, ALS-FTD > ALSpure, ALSci
									bvFTD > ALSbi
									ALSbi > ALSpure
Stereotypic behaviors	15.18 (11.28)	10.16 (5.73)	33.85 (22.53)	35.00 (15.65)	48.76 (27.53)	45.12 (26.22)	30.172^a^	<0.001	bvFTD, ALS-FTD, ALScbi > ALSpure, ALSci
									ALSbi > ALSci
Reduced motivation	12.57 (10.94)	12.50 (7.23)	43.46 (22.02)	49.13 (26.51)	60.55 (30.53)	49.86 (29.08)	46.599^a^	<0.001	bvFTD, ALS-FTD, ALScbi, ALSbi > ALSpure, ALSci

*Means (Standard Deviation)*.

a *Welch's F value*.

*ALSbi, amyotrophic lateral sclerosis with behavioral impairment; ALSci, amyotrophic lateral sclerosis with cognitive impairment; ALScbi, amyotrophic lateral sclerosis with combined cognitive and behavioral impairment; ALSpure, amyotrophic lateral sclerosis with no cognitive or behavioral impairment; ALS-FTD, amyotrophic lateral sclerosis-frontotemporal dementia-motor neuron disease; bvFTD, behavioral-variant frontotemporal dementia; CBI-R, the revised Cambridge behavioral Inventory*.

#### ALS Subgroups

Direct comparison between ALS subgroups revealed more severe stereotypic behavior and reduced motivation in ALScbi compared to both ALSpure (*p* = 0.03 and *p* = 0.016) and ALSci (*p* = 0.006 and *p* = 0.015) groups. Similarly greater extent of reduced motivation was also identified in ALSbi compared to ALSpure and ALSci patients (both *p* = 0.003). ALSbi further demonstrated disproportionate impairment in abnormal behavior compared to ALSpure patients (*p* = 0.032), in stereotypic behavior relative to ALSci patients (*p* = 0.037), as well as in reduced motivation compared to both ALSpure and ALSci groups (both *p* = 0.003). As expected, ALSbi and ALScbi subgroups demonstrated more severe behavioral impairments compared to ALSpure and ALSci patients.

### Non-Motor Disturbances Across Sex, Age, Disease Stage and Onset Site

#### Impact of Sex

Within the ALS subgroups combined (ALSpure, ALSbi, ALSci and ALScbi) there were no significant differences in terms of frequency of non-motor symptoms according to sex (all *p* > 0.45), however when stereotypies and changes in sleep were present they tended to be more severe in males (*M* = 26.7, *SD* = 21.4 and *M* = 48.3, *SD* = 27.6 respectively) than females (*M* = 17.3, *SD* = 10 and *M* = 37.4, *SD* = 20.9 respectively; both *p* = 0.04).

#### Impact of Age

With ALS patients stratified according to the median age at time of assessment into those with young onset (<60 years) and those with late-onset (>60 years) there was no significant differences in terms of frequency of non-motor symptoms across eating changes (34% and 33% respectively, *p* = 0.7), mood (73 vs. 83%, *p* = 0.2), motivation (54 vs. 68%, *p* = 0.2), sleep (67 vs. 83%, *p* = 0.06), stereotypy (40, 49%, *p* = 0.3) or abnormal behavior (50 vs. 56%, *p* = 0.5). When sleep was impaired this was found to be more severe in late onset cases (*M* = 37.3, *SD* = 27.6) compared to early-onset (*M* = 26.6, *SD* = 27.7; *p* = 0.045).

#### Impact of Disease Duration and Stage

**Figure 3:**
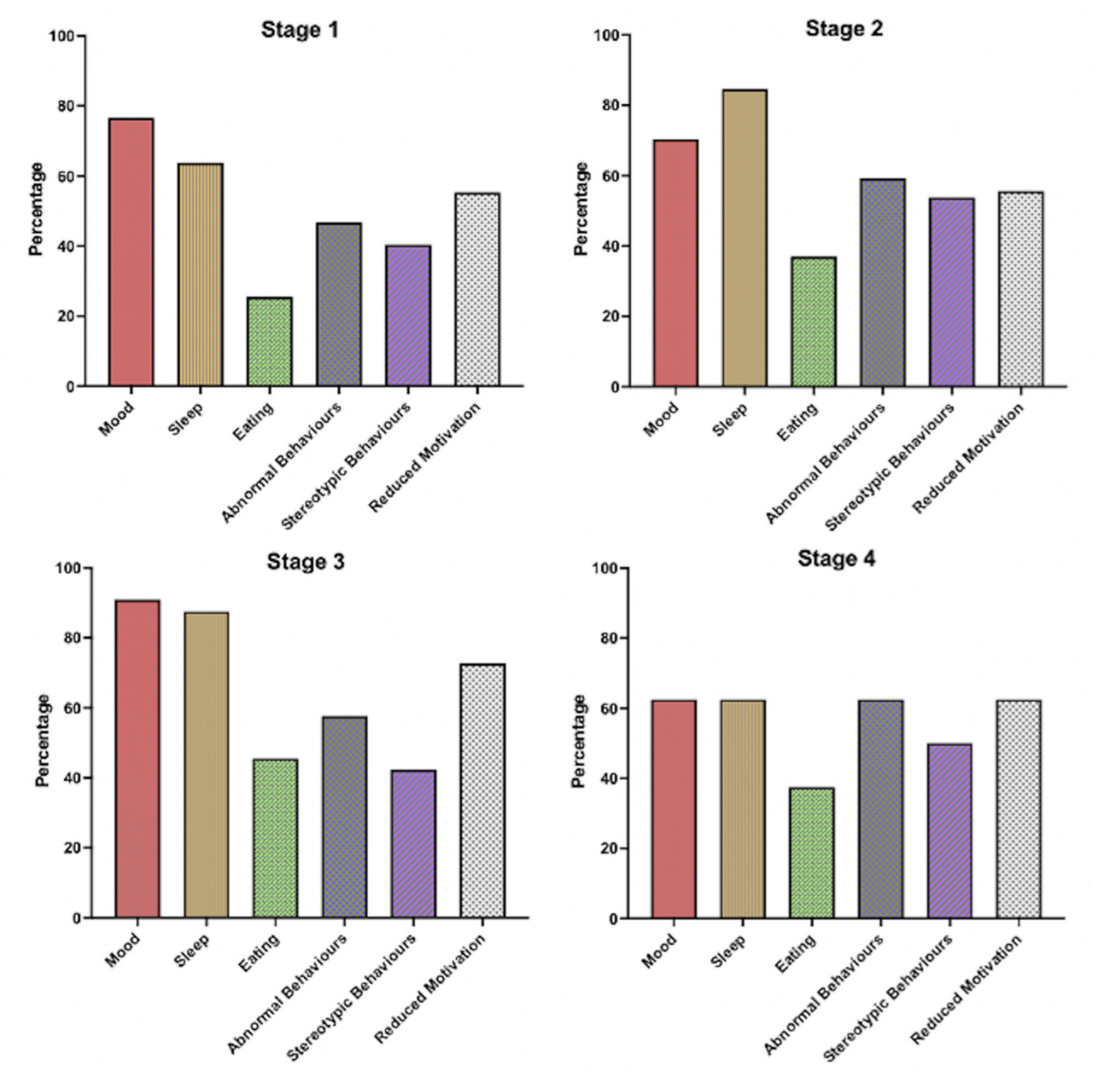
Bar chart displaying frequency of non-motor symptoms within ALS patients stratified by King's Staging Criteria on the revised Cambridge behavioral Inventory (CBI-R).

When ALS patients were stratified by King's staging criteria, no significant differences were revealed in the distribution of mood abnormality, sleep disorder, eating abnormality, stereotypy, apathy and abnormal behavior (*p* > 0.05; see Supplementary Table 1; Figure 3). Specifically, abnormal mood was endorsed by 78% in stage 1, 70% in stage 2, 92% in stage 3 and 60% at stage 4b suggesting that for the majority of people who experience alterations in mood this occurred in the earliest disease stages. A similar pattern was seen for disordered sleep that was present in over 60% of patients in stage 1 and 4b and over 80% in stages 2 and 3. Abnormal behaviors, stereotypic behaviors and reduced motivation were also common at the earliest stage occurring in 48, 40 and 53% respectively that rose marginally at stage 4b to 60, 54 and 60%.

To look at this another way within the confines of a cross-sectional study, we correlated ALSFRS-R with non-motor severity scores and found a negative correlation between ALSFRS-R and severity of (1) stereotypy (*r* = −0.22, *p* = 0.04), (2) mood abnormality (*r* = −0.2, *p* = 0.04), (3), sleep disorder (*r* = −0.21, *p* = 0.03), apathy and abnormal behavior (both *r* = −0.23, *p* = 0.02).

#### Impact of Site of Onset

Site of onset was not found to contribute to the presence of behavioral symptoms, where no significant differences were observed in the distribution of mood abnormality, sleep disorder, eating abnormality, stereotypy, apathy and abnormal behavior between bulbar and limb onset groups (*p* > 0.05; Supplementary Table 2). In terms of effect on severity of behavioral symptoms, bulbar patients demonstrated a significantly higher score in mood symptoms when compared to those with a limb onset (*p* = 0.025; Supplementary Table 3). Sleep disorder, eating abnormality, stereotypy, apathy and abnormal behavior were comparable (*p* > 0.05; Supplementary Table 3).

### Predictors of Overall Severity

Multiple regression analysis revealed that diagnosis group (ALS, ALS-FTD and FTD), disease duration and ACE-III total score together significantly predicted the severity of CBI-R total score, *F*_(5, 211)_ = 12.182, *p* < 0.001, accounting for 20.9% of the variability in overall behavioral disturbance (adjusted *R*^2^ = 0.209). While diagnosis group, longer disease duration and lower ACE-III total score were significantly predictive of higher levels of behavioral abnormalities, education level and *C9orf72* expansion status were not a significant predictor and diagnosis group was found to contribute the most to the variance of behavioral scores (Table 3).

**Table 3:** Regression Coefficients for multiple regression analysis using diagnosis group, disease duration and ACE total score to predict CBI-R total score.

**Variable**	***B***	**SE *B***	**β**	***p***
Diagnosis Group	8.971	0.276	0.276	<0.001
*C9orf72* status	−4.04	−0.063	−0.063	0.31
Disease Duration	0.108	0.198	0.198	0.001
ACE Total Score	−0.192	−0.171	−0.171	0.027
Education (Year)	−0.535	−0.076	0.076	0.234

*B, unstandardized coefficients; SE B, standard error unstandardized coefficients; β, standardized coefficients*.

*ACE, Addenbrooke's Cognitive Examination; CBI-R, the revised Cambridge behavioral Inventory*.

## Discussion

This study compared the patterns of non-motor impairment across the ALS-FTD spectrum and found a strikingly similar pattern of rate and notably, severity of neuropsychiatric impairment across the behavioral phenotypes of ALS and bvFTD, supporting the concept of the ALS-FTD spectrum. Neuropsychiatric symptoms that are considered by definition, to be absent in ALSpure and the cognitive phenotype of ALS, were present in these conditions in a similar pattern to that of the behavioral phenotypes, although they occurred less frequently and were at the milder end of the spectrum. Aside from neuropsychiatric symptoms, disordered sleep and disrupted mood were common across all phenotypes suggesting a shared underlying pathophysiology as a result of the neurodegenerative process. These results suggest that of all non-motor features, sleep dysfunction is most influenced by sex and age—and that respiratory dysfunction alone does not sufficiently account for all changes in sleep as suggested by high rates of sleep disruption across the early disease stages of ALS. Lastly, this study supports the concept that behavioral change occurs early and deteriorates in line with disease progression.

Until now, despite well-documented changes of neuropsychiatric symptoms in ALS, studies exploring the nature and pattern of these changes in comparison to FTD have remained limited. The available studies are subject to the limitations of methodological differences, that in part reflect the recency of criteria for the definition of behavioral and cognitive impairment in ALS, the wide variety of test batteries available to detect neuropsychiatric changes and small numbers of participants. This study had the benefit of large numbers across ALS and FTD phenotypes from a specialist centre with patients classified according to the most recent consensus criteria for behavioral and cognitive impairment in ALS (6). The current findings have revealed a comparable level of disturbance, both in terms of frequency and severity, across stereotypy, loss of motivation and abnormal behavior demonstrated by patients with ALSbi, ALScbi, bvFTD and ALS-FTD. This highlights the critical need for an approach that incorporates behavioral assessments, management strategies and support for patients and carers in multidisciplinary ALS clinics. This also supports the recent consensus criteria that challenged the traditional concept of ALS-FTD as a continuum with ALS at one end of the severity spectrum and bvFTD at the other. The finding of high rates of apathy, although on the milder end of the scale, in patients with ALSpure and ALSci is in keeping with the ALS-FTD spectrum as a more nuanced construct while also reflecting the prevalence of this behavior in ALS, in line with previous findings (3, 12, 31). The pervasiveness of apathy in the ALS phenotypes is presumably in part related to the pathological substrate of apathy, delineated recently as a complex network of cortical and subcortical regions, across the prefrontal cortex, parietal cortex and basal ganglia, that may degenerate early in the ALS-FTD neurodegenerative spectrum (32).

In contrast to apathy, stereotypic and disinhibited behaviors have not received as much intense interest in ALS, despite findings to suggest that behavioral change, and disinhibition in particular, can contribute to dysfunction in activities of daily living (33). Results from the current study suggest that these symptoms are common in the behavioral subtypes of ALS and present with a similar frequency and severity as ALS-FTD and bvFTD. Although generally mild in severity, over 30% of patients categorized as ALSpure and ALSci also exhibited behaviors related to stereotypy and disinhibition. The most comprehensive study of behavior to date in ALS, reported that disinhibition was predominantly related to the *C9orf72* expansion. In this study we showed comparable rates of *C9orf72* expansion across ALS subtypes with no effect on overall behavioral impairment however replication in a larger cohort may identify the impact at an ALS subgroup level. In contrast, disordered eating behaviors has been extensively studied across the spectrum with findings showing a survival benefit in patients with abnormal eating habits and increased BMI, further highlighting the benefit of pursuing detailed exploration of neuropsychiatric symptoms in ALS (34–36). By definition ALSpure and ALSci constitute diagnostic subgroups that do not meet criteria for behavioral impairment, thereby suggesting that neuropsychiatric symptoms may be absent in these cases. Notably, this study identified the presence of the full spectrum of neuropsychiatric symptoms in these cases, albeit classified as mild by carers for the majority. Of note, educational status was lower in ALScbi compared to ALSpure and ALSci that is perhaps relevant in the setting of recent research in ALS suggesting a buffering effect of cognitive reserve on cognitive factors and brain volume (37, 38). In this study educational status was not a significant predictor of overall behavioral impairment suggesting further study is warranted.

Non-Motor impairments are striking in ALSpure, with sleep disturbance present in up to 70% of cases and classified as moderate-severe in almost 40% of cases with similar rates across the spectrum. Disordered sleep was determined by two questions including “*sleep is disturbed at night*” and “*sleeps more by day than usual (cap naps)*” reflecting the typical sleep impairments seen in neurodegeneration of disrupted night-time sleep and disruption of the sleep/wake cycle. Although not a comprehensive assessment of sleep, the results from this study are in line with a recent systematic review on sleep in ALS that identified the prevalence of sleep disturbance as 50–63% based on the Pittsburgh Sleep Quality Index Questionnaire (39). While traditionally disrupted sleep in ALS was assumed to be related to physical disability; cramps and pain due to muscle wasting and immobility; and hypoventilation due to diaphragmatic weakness, more recently the impact of neurodegeneration on the circadian cycle has been implicated as a potential factor (40). In light of the current findings of disturbed sleep across the spectrum of ALS-FTD occurring early in the disease process, it seems possible that neurodegeneration may be a significant driver of sleep disturbance in ALS.

Similarly, mood disorders were prevalent across the entire spectrum of ALS-FTD occurring in over 70% of cases of ALSpure and ALSci and 100% of ALSbi. As with the sleep domain, this did not entail an extensive analysis of mood. Instead, 4 questions were asked regarding whether the patient (1) *cries*, (2) *appears sad or depressed*, (3) *is very restless or agitated and* (4) *is very irritable*. Generally, the prevalence of mood disorders is not understood in ALS with rates ranging from 29% for mild depression to 6% for severe depression, while anxiety been reported in up to 30% of cases—with the suggestion that milder symptoms are most prevalent (41–43). Importantly, these 4 symptoms may be a consequence of other factors outside of depression, including pseudobulbar affect or the grief response. Interestingly, patients with bulbar onset ALS were more likely to experience severe “mood” disorders that may represent an increased rate of pseudobulbar affect as has been previously demonstrated (44). Similarly in FTD, reports have been varied with one meta-analysis reporting a rate of 33% but noted the heterogeneity in reporting measures with differences across caregiver, patient, objective and subjective measures. Another more recent analysis found the rate of depression to be 7–59% and 19–63% for anxiety in bvFTD (45, 46). These results suggest that mood symptoms, but not mood disorders *per se*, may be high across the spectrum.

Previous work has found that cognitive changes within the spectrum of FTD are associated with neuropsychiatric symptoms (47). In the current study, the large number of participants allowed us to explore the effect of various non-modifiable factors on specific behaviors. Of interest was the finding of increased severity of abnormal stereotypic behavior and sleep dysfunction in males across the ALS spectrum of disease. To our knowledge this is the first report of divergence across sex for these non-motor parameters, although there has already been significant interest in sex differences in ALS in terms of prevalence, age of onset and survival (48, 49). Interestingly, the neuroimaging signatures of ALS differs across sex and perhaps consequently, impaired cognition has been described more frequently in males (50, 51). In light of this and given the strong association between cognition and behavior and associated cortical changes, the results from this study are perhaps not surprising. Similarly, age had a negative impact on the severity of sleep dysfunction that may be merely related to the increased population prevalence of sleep disorders including sleep apnoea with increasing age, or may be a factor of disease severity, but nonetheless suggests that clinicians should be alert to severe sleep disorders in older patients.

The findings of non-motor changes early in the disease process is in keeping with previous studies that suggests neuropsychiatric symptoms occur in the early stages of ALS (52). Although this was not a longitudinal study it does suggest that, similar to the current theories that are developing in FTD and are more substantiated in AD, non-motor dysfunction, may occur in the pre-manifest disease stages and may be a useful early diagnostic biomarker (53, 54). These findings also accord with the literature on increased rates of pre-symptomatic psychiatric disorders in ALS with mood symptoms endorsed by almost 80% of carers in this study in King's stage 1 (55). Overwhelmingly, all of the non-motor and neuropsychiatric symptoms were negatively correlated with the functional rating measure, the ALSFRS-R. This further consolidates the concept that behavior deteriorates in line with disease stage in ALS, and expands on this by considering individual symptoms to show that the deterioration does not differentiate across non-motor features. As such it is likely that non-motor symptoms are an integral component of the neurodegenerative process and their progression over time reflects the increasing cortical burden of disease.

As mentioned, although this study was not primarily designed as a longitudinal study the large number of participants allowed us to explore the development and trajectory of neuropsychiatric and non-motor symptoms in ALS and our findings are in line with previous studies. That neuropsychiatric features are common in the behavioral phenotypes of the ALS-FTD spectrum may be considered a somewhat circular argument given that these symptoms are the cardinal features of the disorders. However, the aim of this study was not to identify the behaviors but instead to advance knowledge of the overlap and to validate the concept of the ALS-FTD spectrum that has recently been challenged. By comparing the pattern and, in particular, the severity across the spectrum we sought to identify predictors that could alert clinicians to the presence of non-motor impairment. Further dedicated longitudinal studies may advance knowledge into these behaviors and how they change over time, to improve prediction of disease progression and draw distinctive comparisons with other neurodegenerative conditions. Further neuroimaging analysis might also be incorporated into future studies to understand associations between structural changes of the brain and disease stage.

The use of the ACE-III as opposed to an ALS-specific measure such as the ECAS, as a measure of general cognition should also be recognized as a limitation in this study. In this and previous published studies from our research group, where this unique cohort across FTD and ALS have been recruited, we have selected to use the ACE-III as a measure of general cognition to allow comparison across all disease groups (3, 56). While including a full battery of neuropsychological assessments was beyond the scope of this study in which the goal was to categorize patients according to current consensus criteria, its inclusion in future studies might identify subtle and more selective cognitive abnormalities that may influence patient classification. Due to the constraints of using a carer-based measure the current data does not allow us to compare the rates of non-motor impairment to that of the healthy population. Future study design should consider this issue in the selection of measures particularly in the context of sleep and mood.

In summary, patient and carer experiences of common neuropsychiatric symptoms and non-motor symptoms of mood and sleep dysfunction associated with ALS has an undisputed role in how we diagnose and classify ALS and evaluate disease progression. Through awareness and understanding, patients and their caregivers can be informed that these behaviors are a part of the disease process and not a product of dysfunction within their relationships. The findings from this study suggest that non-motor features and particularly neuropsychiatric features can be severe in patients with ALS and that mood and sleep disorders are inherent across all subtypes of disease. This study also suggests a high index of suspicion for more severe behavioral impairment in males and has confirmed that behavioral symptoms occur early. Awareness and identification of non-motor features are an important aspect of formulating treatment plans, managing the disease and educating all groups affected by cognitive and behavioral impairments associated with ALS. Collectively, this suggests a role for an expanded multidisciplinary team that includes cognitive and behavioral professionals for the management of ALS.

## Data Availability Statement

The raw data supporting the conclusions of this article will be made available by the authors, without undue reservation.

## Ethics Statement

The studies involving human participants were reviewed and approved by the South Eastern Sydney Local Health District, the University of New South Wales, and the University of Sydney Ethics Committees. The participants or the participants legal guardian/next of kin provided written informed consent to participate in this study.

## Author Contributions

ED: study concept, clinical phenotyping, data analyses, and manuscript drafting and preparation. KM: study concept, data analyses, and manuscript drafting and preparation. NT, JC, and OP: data analyses and manuscript drafting and preparation. TD, WH, CM, MZ, and RA: clinical phenotyping and manuscript drafting and preparation. JH: clinical phenotyping, data analyses, and manuscript drafting and preparation. MK: study concept, clinical phenotyping, and manuscript drafting and preparation. All authors contributed to the article and approved the submitted version.

## Funding

This work was supported by funding to Forefront, a collaborative research group dedicated to the study of Frontotemporal Dementia and Motor Neuron Disease, from the National Health and Medical Research Council of Australia (NHMRC) program grant (#1037746, #1132524), dementia team (#1095127) grants) and the Australian Research Council Centre of Excellence in Cognition and its Disorders Memory Program (#CE110001021). ED is supported by a NHMRC postdoctoral fellowship (#APP117858). CD-S is supported by a NHMRC Boosting Dementia Research Leadership Fellowship (#1138223). JK is supported by NHMRC Dementia Research Team Grant (#APP1095127). GMH is a NHMRC Leadership Fellow (#1176607). RA is supported by a NHMRC post-doctoral fellowship. TD is supported a NHMRC post-doctoral fellowship (#APP1162075). OP is supported by a NHMRC Senior Research Fellowship (APP1103258). MK is supported by NHMRC Partnership Project (#1153439) and a NHMRC Practitioner Fellowship (1156093).

## Conflict of Interest

The authors declare that the research was conducted in the absence of any commercial or financial relationships that could be construed as a potential conflict of interest.

## Publisher's Note

All claims expressed in this article are solely those of the authors and do not necessarily represent those of their affiliated organizations, or those of the publisher, the editors and the reviewers. Any product that may be evaluated in this article, or claim that may be made by its manufacturer, is not guaranteed or endorsed by the publisher.
