# Supplementary material for: Factors That Influence Non-Motor Impairment Across the ALS-FTD Spectrum: Impact of Phenotype, Sex, Age, Onset and Disease Stage
Source: Front Neurol. 2021 Nov 25;12:743688. doi: 10.3389/fneur.2021.743688 (PMC8656429; doi:10.3389/fneur.2021.743688)
Supplement: Supplementary file 1 [file Data_Sheet_1.docx]

**Supplementary Table 1.** Prevalence of non-motor symptoms within ALS patients stratified by King’s Staging Criteria

| **King’s staging** | **Stage 1 (*n* = 47)** | **Stage 2**  **(*n* =27)** | **Stage 3**  **(*n* =33)** | **Stage 4**  **(*n* =8)** | **Chi-square value** | ***p*** |
| --- | --- | --- | --- | --- | --- | --- |
| Mood Changes | 76.6% | 70.4% | 90.9% | 62.5% | 5.336 | .149 |
| Sleep Changes | 63.8% | 84.6% | 87.5% | 62.5% | 7.786 | .051 |
| Eating Changes | 25.5% | 37.0% | 45.5% | 37.5% | 3.516 | .319 |
| Abnormal behaviours | 46.8% | 59.3% | 57.6% | 62.5% | 1.681 | .641 |
| Stereotypic behaviours | 40.4% | 53.8% | 42.4% | 50.0% | 1.387 | .709 |
| Reduced motivation | 55.3% | 55.6% | 72.7% | 62.5% | 2.885 | .410 |

ALS = amyotrophic lateral sclerosis.

**Supplementary Table 2.** Prevalence of non-motor symptoms within ALS patients stratified by site of onset

|  | **Bulbar (*n* = 47)** | **Limb (*n* =68)** | **Chi-square value** | ***p*** |
| --- | --- | --- | --- | --- |
| Mood Changes | 87.2% | 72.1% | 3.762 | .052 |
| Sleep Changes | 72.3% | 77.3% | .358 | .549 |
| Eating Changes | 40.4% | 30.9% | 1.116 | .291 |
| Abnormal behaviours | 59.6% | 36.7% | 1.025 | .311 |
| Stereotypic behaviours | 47.8% | 30.4% | .298 | .585 |
| Reduced motivation | 66.0% | 57.4% | .864 | .353 |

ALS = amyotrophic lateral sclerosis.

**Supplementary Table 3.** Severity of neuropsychiatric and non-motor symptoms between limb and bulbar onset within ALS patients

|  | **Bulbar (*n* = 47)** | **Limb (*n* =68)** | ***t*** | ***p*** |
| --- | --- | --- | --- | --- |
| Mood Changes | 24.73(20.40) | 17.07(15.78) | 2.271 | .025 |
| Sleep Changes | 35.37(31.96) | 30.30(26.04) | .927 | .356 |
| Eating Changes | 9.84(15.62) | 5.33(10.34) | 1.733 | .087 |
| Abnormal behaviours | 11.08(16.33) | 6.68(9.55) | 1.662^a^ | .101 |
| Stereotypic behaviours | 10.33(16.42) | 10.02(16.56) | .098 | .922 |
| Reduced motivation | 16.12(20.23) | 13.07(21.24) | .771 | .442 |

Means (Standard Deviation). ^a^Equal variance not assumed.

ALS = amyotrophic lateral sclerosis.
